# In vivo polyester immobilized sortase for tagless protein purification

**DOI:** 10.1186/s12934-015-0385-3

**Published:** 2015-11-25

**Authors:** Iain D. Hay, Jinping Du, Patricia Rubio Reyes, Bernd H. A. Rehm

**Affiliations:** Institute of Fundamental Sciences, Massey University, Palmerston North, New Zealand; Department of Microbiology, Monash University, Clayton, 3800 Australia; MacDiarmid Institute for Advanced Materials and Nanotechnology, Wellington, New Zealand; Polybatics Ltd., Palmerston North, New Zealand

**Keywords:** Protein purification, Polyhydroxyalkanoate, Sortase, Self-cleavage

## Abstract

**Background:**

Laboratory scale recombinant protein production and purification techniques are often complicated, involving multiple chromatography steps and specialized equipment and reagents. Here it was demonstrated that recombinant proteins can be expressed as covalently immobilized to the surface of polyester (polyhydroxyalkanoate, PHA) beads in vivo in *Escherichia coli* by genetically fusing them to a polyester synthase gene (*phaC*). The insertion of a self-cleaving module, a modified sortase A (SrtA) from *Staphylococcusaureus* and its five amino acid recognition sequence between the synthase and the target protein led to a simple protein production and purification method.

**Results:**

The generation of hybrid genes encoding tripartite PhaC-SrtA-Target fusion proteins, enabled immobilization of proteins of interest to the surface of PHA beads in vivo. After simple cell lysis and isolation of the PHA beads, the target proteins could be selectively and efficiently released form the beads by activating the sortase with CaCl_2_ and triglycine. Up to 6 mg/l of soluble proteins at a purity of ~98 % could be isolated in one step with no optimization. This process was used to produce and isolate three proteins: Green fluorescent protein, maltose binding protein and the *Mycobacteriumtuberculosis* vaccine candidate Rv1626.

**Conclusions:**

We have developed a new technique for easy production and purification of recombinant proteins. This technique is capable of producing and purifying high yields of proteins suitable for research application in less than 2 days. No costly or specialized protein chromatography equipment, resins, reagents or expertise are required.

**Electronic supplementary material:**

The online version of this article (doi:10.1186/s12934-015-0385-3) contains supplementary material, which is available to authorized users.

## Background

The expression and purification of a target protein of interest is a common undertaking in many research laboratories. This task is often complicated by the multiple chromatography steps required to obtain a product of acceptable purity. Furthermore, most lab-scale purification techniques require an affinity tag such as His, Strep, or GST tag to be engineered into the protein [1]. These tags require specific and often costly chromatography resins to isolate the target protein. Where tags may affect the structure, function or immunogenicity of the target protein (e.g. protein crystallography or antigens for antibody generation), they may need to be removed after purification by engineering a site-specific protease recognition site between the tag and the target protein. To remove the tag the purified protein is treated with a site-specific protease such as TEV protease; the released tag and added protease must then be removed from the sample by further chromatography steps [2]. This introduces additional steps to the workflow, decreasing the yield, and increases the risk of protein loss/degradation. Furthermore, the cleavage can often result in a “scar” (additional residual amino acids) which may negatively impact the target protein.

An alternative approach is to utilize a self-cleaving affinity tag. This approach utilizes a hybrid module composed of an affinity tag and an auto-processing protease or a modified intein. The target protein is genetically fused to this module and bound to the affinity resin, the auto-cleaving reaction can be activated by the addition of a cofactor (e.g. metal ions) or a shift in temperature or thiol for intein auto processing. The target protein can be eluted from the resin while the self-cleaving affinity tag remains bound [3]. An advantage of this technique is that many of these auto-processing domains result in no or minimal scars on the target protein after cleavage.

A modified form of the well-characterized cell surface sortase transpeptidase A (SrtA) from *Staphylococcus* *aureus* [4] represents one of such auto processing modules. In Gram-positive bacteria sortase proteins are responsible for linking specific secreted proteins to the cell wall peptidoglycan. SrtA does this by recognising a five amino acid “sorting signal” in the target protein, cleaving this signal and linking the C-terminus of the protein to pentaglycine in the cell wall. A soluble form of SrtA has previously been modified to self-cleave in the presence of Ca^2+^ by including the sorting signal on its C-terminus. By fusing a His tag to the N-terminus and a protein of interest immediately after the sorting signal a simple purification technique was developed [5, 6]. Cleavage occurs between T and the G of the sorting signal leaving a single additional G on the N-terminus of the C terminally fused target protein.

**Fig. 1:**
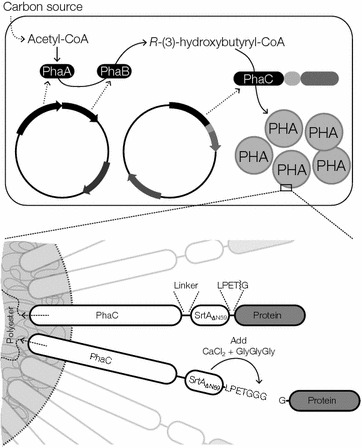
Schematic representation of the PHA immobilized sortase protein production and purification method described in this study

Here we utilized the ability to covalently immobilize proteins to the surface of bacterially produced polyester beads in vivo. This approach exploits the ability to genetically fuse proteins or protein domains to the PHA synthase from *Ralstonia* *eutropha* (PhaC). When the PHA synthase is supplied with *R*-(3)-hydroxybutyryl-CoA by the PhaA and PhaB enzymes it produces an insoluble polyester in the form of polyhydroxyalkanoate inside the cell. The PHA synthase remains covalently attached to the nascent PHA chain, which self-assembles into beads in the bacterial cytosol with a diameter of 100–500 nm. When the synthase is overexpressed in *Escherichiacoli* it densely coats the surface of these beads [7, 8]. This technique has been employed to display various proteins and domains on the surface of PHA beads [9–14]. By combining this technique with the sortase based auto-cleavage process described above (replacing the His tag with the PHA synthase) we remove the requirement to use costly affinity resins, as the fusion protein is covalently attached to PHA beads that can be easily isolated from the cell (Fig. 1).

We demonstrated the practicality of this technique by recombinantly expressing and purifying three proteins at laboratory scale. This method can produce high yields of proteins without the need to modify the protein with cumbersome affinity tags. The proteins were purified to a level adequate for most common research laboratory needs without the need for specialised protein purification equipment or expertise.

## Results and discussions

### Sortase transpeptidase from *S.* *aureus* can be functionally immobilized on PHA beads

To first assess whether the sortase A transpeptidase for *S.* *aureus* (SrtA) could be functionally immobilized on the surface of PHA beads in vivo we made genetic fusions of the soluble (non-membrane anchored) form of SrtA (SrtAΔN59) [15] to the C-terminus of PhaC from *Ralstonia* *eutropha*. A “linker” (VLAVIDKRGGGGG) is included between the two proteins to allow them to fold and function independently [12]. When this hybrid gene was introduced into *E.* *coli* strains harboring the plasmid pBBRMCS69 (containing *phaA* and *phaB* to provide the *R*-(3)-hydroxybutyryl-CoA substrate for PhaC PHA synthase) it mediated production of similar yields of both PHA and fusion protein when compared to the unmodified PhaC gene (data not shown).

**Fig. 2:**
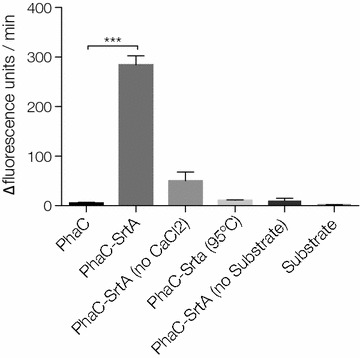
Activity of the PhaC-SrtA PHA beads measured via cleavage of the synthetic sortase FRET substrate. Fluorescent units are arbitrary. ***p = 0.0006

To assess the peptidase activity of the immobilized SrtA we employed a synthetic fluorescently self-quenched peptide FRET substrate, composed of the fluorophore (EDANS) and a quencher (DABCYL) separated by the 5 amino acid *S.* *aureus* sorting signal (LPETG). If the sorting signal is cleaved then the fluorophore is separated from the quencher and its fluorescence can be detected. The substrate was added to a suspension of the PHA beads and incubated. No significant activity could be detected from the PhaC beads, whereas significant activity could be detected from the PhaC-SrtA beads (Fig. 2). The activity was dependent on the presence of CaCl_2_ and could be removed by denaturing the protein on the beads at 95 °C for 15 min before conducting the assay, indicating that this activity is the result of the immobilized sortase.

### Proof of concept—Tripartite PhaC-SrtA-Target fusions can be used for recombinant protein production and purification

To utilize the immobilized sortase beads for recombinant protein production and purification we generated two tripartite fusion protein encoding hybrid genes to prove the concept of this recombinant protein purification technique. The fusion proteins were designed to have SrtA fused to the C terminus of PhaC as above. A LPETG sorting signal was included on the C terminus of SrtA, the target protein would then be fused immediately after the sorting signal.

Green fluorescent protein (GFP) and maltose binding protein (MBP) were used as target proteins for proof of concept experiments probing the utility PhaC-SrtA fusions attached to PHA beads for the production and purification of recombinant proteins. Tripartite PhaC-SrtA-GFP or MBP translational fusions were generated. In order to minimize the scar on the target protein an *Age*I restriction site was used to ligate the target gene to SrtA (an in-frame *Age*I—ACCGGT—site encodes the amino acids TG in the LPETG sorting signal). The sortase function of the fusion protein cleaves between the T and the G of the sorting signal and thus the target protein will be released with a single additional G on its N-terminus.

Although *E.* *coli* has low cytosolic levels of free Ca^2+^ (generally reported to be maintained in the range of 100–300 nM [16, 17]), we modified the previously reported growth and PHA isolation process [18] to minimize the risk of premature autoprocessing of the fusion protein during the growth phase: Cells were grown in Terrific broth at 37 °C for 16 h instead of in LB with glucose at 25 °C for 48 h as previously reported; EGTA was added to the lysis and wash buffers to chelate any extracellular Ca^2+^ which may otherwise activate the sortase; The cells were lysed with commercial blend of detergents (BugBuster^®^, Novagen), and sonication. The reported glycerol gradient ultracentrifugation methods was replaced by washing in Tween 20 with sonication steps. This resulted in faster lysis and isolation of fusion protein displaying PHA beads and removed the need for specialized equipment (ultracentrifuge).

**Fig. 3:**
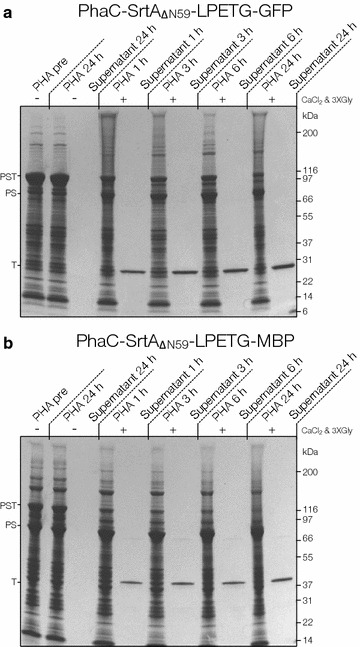
Purification of GFP (A), MBP (B), and RV1622 from the PhaC-SrtA-Target beads. SDS-PAGE of the PHA bead and soluble fraction before and after activation with CaCl_2_ and triglycine at different time points. *PST* PhaC-SrtA-Target band (pre cleavage); *PS* PhaC-SrtA band (post cleavage); *T* target protein

Both the PhaC-SrtA-GFP and the PhaC-SrtA-MBP fusion mediated cell densities and PHA yields similar to that mediated by unmodified PhaC (Table 1). PHA beads could be isolated from both strains producing the respective fusion protein and a dominant protein corresponding to the PhaC-StrA-Target could be detected attached to the beads by SDS-PAGE (PST band Fig. 3). Little premature cleavage (PS band PhaC-SrtA) could be detected with GFP as target protein (Fig. 3a) but moderate levels could be detected with MBP target protein (Fig. 3b). Sortase A requires Ca^2+^ for hydrolysis activity and an oligo-glycine nucleophile for optimal kinetics [19]. Thus the sortase could be activated by washing the EGTA off the beads and by adding CaCl_2_ and triglycine. When CaCl_2_ and triglycine are not added to the buffer no released target protein (GFP or MBP) could be detected and the PhaC-SrtA-Target fusion protein stays intact and attached to beads after 24 h of incubation. Whereas when CaCl_2_ and triglycine were included in the buffer >60 % of the available target protein was released within 1 h (Fig. 3). An increase in target product and a shift of the PhaC-SrtA-Target (PST) band to the PhaC-SrtA (PS) band could be observed over 6 h. In both cases prolonged incubation for 24 h resulted in higher yields of the target protein (T) and almost complete conversion of PhaC-SrtA-Target into PhaC-SrtA plus the soluble target protein (Fig. 3). In both cases little to no contaminating proteins were observed in the supernatants on SDS-PAGE (Fig. 3). Identities of the target protein bands were confirmed by MALDI-TOF/MS analysis of tryptic peptides. Densitometry indicates the target proteins were present in the supernatant in >98 % purity in both cases.

**Table 1:** Yield and binding capacity of the proof of concept PhaC-SrtA-Target PHA beads

Construct	mg target protein eluted per g wet bead	MW of target (g/mole)	nmoles target per g wet bead	g Wet beads/L	mg target protein/L	nmoles protein/L	g dry cells/L	% PHA by GCMS
PhaC-Srt-LPETG-GFP	2.84	26,820	106.03	2.14	6.08	226.9	3.870	27.50
PhaC-Srt-LPETG-MBP	2.23	40,254	55.29	2.24	5.00	123.85	3.740	20.66
PhaC	0.00	–	–	2.68	–	–	3.488	30.72

For the GFP fusion protein 2.84 mg of target GFP could be released from 1 g of wet beads, this corresponds to 106.0 nmoles of GFP per gram of wet beads. The MBP fusion protein beads released 2.23 mg (55.3 nmoles) of MBP per 1 g of wet beads. In lab scale shake flasks this corresponds to 6.08 mg (226 nmoles) and 5.00 mg (123.85 nmoles) of purified target protein (GFP and MBP respectively) produced per liter without optimization (Table 1). Fluorescence from the GFP on the un-activated beads and in the purified supernatant could be detected, and all the MBP from the supernatant could be bound to an amylose column (Additional file 1: Figure S1) suggesting that these proteins were produced in a functional form.

This method produces high yields of soluble proteins without the need to modify the protein with affinity tags that can negatively affect the structure or function of the target protein. Target proteins are produced with a single G scar on the N-terminus; G’s properties (uncharged and the smallest possible amino acid) should make it a relatively innocuous addition to most proteins. The proteins are purified to a level adequate for most common research laboratory needs and can be produced and isolated without the need for specialised or costly protein purification/chromatography equipment, reagents, resins or expertise. All equipment and reagents required are commonly found in most life science laboratories. For downstream use, proteins can be easily rebuffered/dialysed to remove the CaCl_2_ and triglycine. The production and isolation of a soluble highly purified target protein can be completed in less than 2 days with this method.

Grage et al. [20] used PhaC engineering for target protein purification by translationally fusing the target protein to the N-terminus of PhaC including an enterokinase cleavage site as linker. Enterokinase cleavage of respective beads resulted in release of the pure soluble target protein. However, the costly use of the enterokinase and the low cleavage efficiency of about 20 % made this approach less attractive. In another approach two PHA surface proteins: the PhaF phasin and the regulatory protein PhaR were used in combination with intein mediated self-cleavage module for protein release [21–23]. However, these methods had two main drawbacks: the binding between the phasin/PhaR and the PHA beads are less stable non-covalent interactions, and the lack to tightly control the intein mediated cleavage in vivo and once the beads are isolated. Phasins bind to the hydrophobic surface of the PHA and other hydrophobic plastics and the binding is dependent on the salt concentration of the buffer, typically when eluting proteins from a resin one uses a relatively high salt concentration to avoid other proteins non-specifically binding to the resin. Yet under high salt concentrations the phasin can dissociate from the PHA beads and result in contamination of the target protein. Under low salt concentrations the phasin is tightly bound to the PHA beads but non target proteins also loosely bind to the PHA beads and can be present in the elution supernatant [21]. In the present system the PhaC PHA synthase is used as a scaffold, PhaC remains covalently attached to the PHA via a thioester linkage as it is synthesized [7], thus the fusion protein remains attached to the beads regardless of salt concentrations. Intein mediated cleavage reactions typically involve activation by a reducing agent such as DTT (which can disrupt native disulphide bonds in the target protein) or relatively small pH changes (lowering the pH below 7.0), thus the fusion protein can not be in environments with a pH below approximately 7. The pH of the *E.* *coli* cytosol is reported to be in the range of 7.2–7.8 and can be influenced during growth by the extracellular environment particularly high levels of acetate [24]. As media acidification and acetate production are common by-product of lab scale bacterial fermentation [25] the premature in vivo and post lysis activation of inteins is a common problem [3], indeed significant premature cleavage was present in all three examples of the phasin/phaR-intein method [21–23]. By using the tighter control of the SrtA auto-processing module instead of inteins for the cleavage reaction the premature cleavage and target loss can be minimized.

An additional benefit comes from the physical immobilization of the proteins during growth. It is widely accepted that the immobilization of proteins can have a beneficial effect on the stability and solubility of proteins, thus the immobilisation of recombinant fusion proteins on the surface of PHA beads in vivo may aid in the functional folding of difficult proteins (i.e., those prone to inclusion body formation). Furthermore, the N-terminal SrtAc tag has been found to enhance the solubility and stability of its fusion partner [5].

### Extending the technique to produce and purify real-world targets

To further assess the broad applicability of this method to produce and purify a range of different proteins we targeted the antigen RV1626 which is an emerging *Mycobacterium* *tuberculosis* vaccine candidate [26, 27]. The PhaC-SrtA-RV1626 fusion-protein expressing plasmid was generated as above. The proteins was produced and isolated as above with minor modifications. The RV1626 containing fusion protein was produced in ClearColi BL21(DE3) *E.* *coli* cells (has proprietary mutations in LPS genes which eliminates the immunogenic endotoxin) to minimize the endotoxin in the final antigen product.

**Fig. 4:**
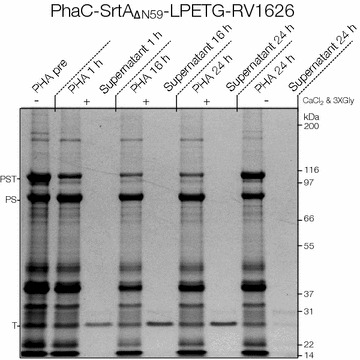
Purification of RV1626 from the PhaC-SrtA-Target beads. SDS-PAGE of the PHA bead and soluble fraction before and after activation with CaCl_2_ and triglycine at different time points. *PST* PhaC-SrtA-Target band (pre cleavage); *PS* PhaC-SrtA band (post cleavage); *T* target protein

PHA beads could be isolated from the respective recombinant bacteria and the PhaC-SrtA-Target fusion protein could be detected as the dominant protein on the beads (Fig. 4). Levels of premature cleavage (PhaC-Srt) were similar to that of the MBP displaying PHA beads. Activation of the beads with CaCl_2_ and triglycine resulted in 0.407 mg (17.95 nmoles) of Rv1626 protein per gram of wet PHA beads after 24 h or 2.04 mg (89.99 nmoles) Rv1626 per L culture.

## Conclusions

Here it was demonstrated that the ability to functionally co-immobilize Sortase A from *S.* *aureus* together with a protein of interest on the surface of PHA beads in vivo enabled the specific cleavage/release of the target protein i.e., production and purification of the protein of interest.

Sortase A from *S.* *aureus* could be functionally immobilized in vivo to the surface of PHA beads. These beads could be used to cleave the LPETG peptide. This activity was used to design a system for recombinant protein production and their tag free purification. The recombinant protein of interest is genetically fused to the PhaC-SrtA fusion protein via a sortase recognition site. The protein is produced as immobilized on the surface of PHA beads in vivo. The respective beads can be easily isolated and the protein released from the beads with the addition of CaCl_2_ and triglycine. As the PHA production itself has been established as commercially scalable process it provides the foundation for scalable and industrial PHA bead-based protein production [8]. After protein release PHA could be recovered from beads as added value. The plasmids required for this technique are generated by a simple restriction enzyme cloning step. This technique requires no costly protein purification or chromatography equipment or expertise and no additional resin or expensive/uncommon chemicals are required. The soluble target proteins can be functionally isolated, without an extensive tag, at high concentration at a purity of up to 98 % in a single step. This is adequate for many common life science research applications including protein production for functional analysis, protein crystallography, or antigen generation for antibody generation.

## Methods

### Bacterial strains and growth conditions

All *E.* *coli* strains and plasmids used in this study are listed in Additional file 2: Table S2. Strains were grown in Terrific Broth (12 g/l tryptone, 24 g/l yeast extract, 4 ml/l glycerol, 17 mM KH_2_PO_4_, 72 mM K_2_HPO_4_) at 37 °C. Where required, antibiotics were used at the following concentrations: ampicillin, 75 μg/ml; chloramphenicol, 34 μg/ml. For PHA bead and protein production, *E.* *coli* strains were first transformed with the PhaA and PhaB encoding plasmid pMCS69 [28], and subsequently with the fusion protein expressing plasmid. Cultures were grown in TB with 37 °C to an optical density at 600 nm (OD_600_) of 0.4–0.5, induced with 1 mM IPTG (isopropyl-β-d-thiogalactopyranoside), and allowed to grow for approximately 16 h.

### Plasmid construction

The PhaC-SrtA expressing plasmid (pET14:PhaC-SrtA) was constructed as follows: The srtA gene minus the N-terminal 59 amino acid membrane anchor region flanked by XhoI and BamHI sites was synthesized by Genscript, the product was cleaved with *Xho*I and *Bam*HI and ligated into the corresponding sites on the plasmid pET14b:phaC-linker-MalE [12].

To make the tripartite fusion protein expressing plasmids (pET14:PhaC-SrtA-GFP, pET14:PhaC-SrtA-MBP, pET14:PhaC-SrtA-RV1626) the srtA_ΔN59_ region was amplified from pET14:PhaC-SrtA with the primers SrtAN59_F and SrtAΔTAA_LPETG_R—this product does not have a stop codon and has LPETG coding region added on the C-terminus (with the TG encoded by an *Age*I site). This was ligated into pGEMteasy according to manufactures instructions. The GFP, MBP, and RV1626 coding regions were synthesized by Genscript or amplified from existing sources with the start codon replaced with an *Age*I site and a *Bam*HI site after the stop codon. The target genes were ligated into the *Age*I and *Bam*HI sites of the pGEMteasy-SrtA plasmid described above. The srtA-target genes were cut from the pGEMteasy backbone with *Xho*I and *Bam*HI and ligated into the corresponding sites on the plasmid pET14b:phaC-linker-MlaE.

### PHA bead isolation

Cells were harvested by centrifugation at 6000 g and washed once in TBS (50 mM Tris–Cl, pH 7.8, 150 mM NaCl) with 10 mM EGTA. Cell pellets were suspended in 1/3 culture volume TBS, 10 mM EGTA, 50 μg/ml lysozyme, 10 μg/ml DNase, 1X Bugbuster (Novagen) and incubated for 15 min at room temperature after which they were sonicated (20 on 20 s off) for a total of 1 min sonication. After lysis the insoluble PHA material was collected by centrifugation at 6000*g* and resuspended in fresh TBS, 10 mM EGTA, 0.05 % Tween20 with brief sonication. This washing step was repeated twice. Finally the beads were resuspended to a 20 % slurry in TBS, 10 mM EGTA, 0.05 % Tween20.

### Activation of sortase beads and isolation of target protein

To activate the sortase beads the slurry was centrifuged at 6000*g* and the pellet resuspended to a 20 % slurry in TBS, 0.05 % Tween20, 5 mM CaCl_2_, 10 mM Gly-Gly-Gly. The beads were incubated on a rotary mixer at 37 °C for 1–24 h. To isolate the released soluble target protein the mixture was centrifuged at 13,000*g* for 10 min and the supernatant analyzed by SDS-PAGE.

### Sortase A assay

To assess the function of the PhaC-SrtA beads a synthetic DABCYL-LPETG-EDANS substrate (Anaspecwas used). The peptide was dissolved in DMSO and added at a final concentration of 5 μM to a 5 % slurry of beads in TBS with 5 mM CaCl_2_. Fluorescence was monitored over time using a FLUOstar Omega (BMG labtech) microplate reader with the sample shaking between readings.

## Additional files

10.1186/s12934-015-0385-3 (A) GFP fluorescence can be detected on the PhaC-SrtA-GFP beads and retains its fluorescence once cleaved from the beads. Beads or purified proteins were placed on a UV transilluminator and imaged. (B) MBP produced and purified with on the PhaC-SrtA-MBP beads is functiona. The purified supernatant was applied to an amylose resin and eluted with maltose indicating the maltose binding function of the protein was retained.
10.1186/s12934-015-0385-3 A list of all *E.* *coli* strains and plasmids used in this study.

## Abbreviations

PHA: polyhydroxyalkanoate
GFP: green fluorescent protein
MBP: maltose binding protein
IPTG: isopropyl β-d-1-thiogalactopyranoside
DABCYL: 4-((4-(dimethylamino)phenyl)azo)benzoic acid
EDANS: 5-((2-Aminoethyl)amino)naphthalene-1-sulfonic acid
EGTA: ethylene glycol tetraacetic acid
FRET: Förster resonance energy transfer
