# Supplementary figures and images for: In vivo polyester immobilized sortase for tagless protein purification
Source: Microb Cell Fact. 2015 Nov 25;14:190. doi: 10.1186/s12934-015-0385-3 (PMC4658790; doi:10.1186/s12934-015-0385-3)

A

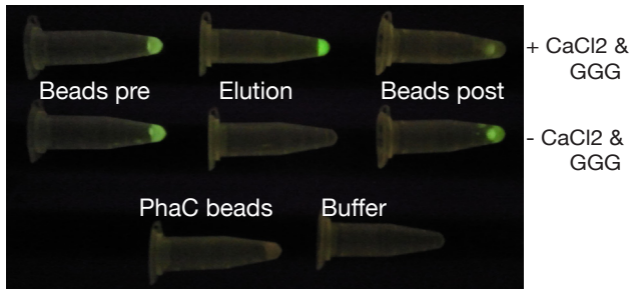

B

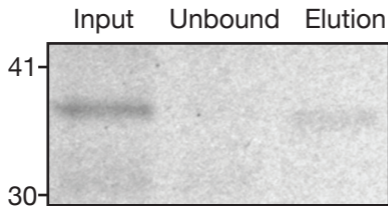

Supplement: Supplementary file 1 — 10.1186/s12934-015-0385-3 (A) GFP fluorescence can be detected on the PhaC-SrtA-GFP beads and retains its fluorescence once cleaved from the beads. Beads or purified proteins were placed on a UV transilluminator and imaged. (B) MBP produced and purified with on the PhaC-SrtA-MBP beads is functiona. The purified supernatant was applied to an amylose resin and eluted with maltose indicating the maltose binding function of the protein was retained. [file 12934_2015_385_MOESM1_ESM.pdf]
