# Supplementary material for: In vivo polyester immobilized sortase for tagless protein purification
Source: Microb Cell Fact. 2015 Nov 25;14:190. doi: 10.1186/s12934-015-0385-3 (PMC4658790; doi:10.1186/s12934-015-0385-3)
Supplement: Supplementary file 2 — 10.1186/s12934-015-0385-3 A list of all E. coli strains and plasmids used in this study. [file 12934_2015_385_MOESM2_ESM.pdf]

Supplementary Table 1

Strain/Plasmid	Description	Reference
E. coli BL21 DE3	F^- *ompT hsdSB* (rB^- mB^-) *gal dcm* (DE3)	Novagen
E. coli ClearColi	*ΔgutQ ΔkdsD ΔlpxL ΔlpxM ΔpagP ΔlpxP ΔeptA. msbA148.* Modified LPS with no endotoxic response	Lucigen
pMCS69	pBBR1MCS with phaA and phaB	[1]
pETC	pET14b containing wild-type *phaC*	[2]
pET14:PhaC-linker-MalE	pET14b encoding a PhaC-linker-MalE fusion protein	[3]
pET14:PhaC-SrtA	pET14b encoding a PhaC-linker-$SrtA_{ΔN59}$ fusion protein	This study
pET14:PhaC-SrtA-GFP	pET14b encoding a PhaC-linker-$SrtA_{ΔN59}$-LPETG-GFP fusion protein	This study
pET14:PhaC-SrtA-MBP	pET14b encoding a PhaC-linker-$SrtA_{ΔN59}$-LPETG-MBP fusion protein	This study
pET14:PhaC-SrtA-RV1626	pET14b encoding a PhaC-linker-$SrtA_{ΔN59}$-LPETG-RV1626 fusion protein	This study

1. Amara AA, Rehm BH: **Replacement of the catalytic nucleophile cysteine-296 by serine in class II polyhydroxyalkanoate synthase from Pseudomonas aeruginosa-mediated synthesis of a new polyester: identification of catalytic residues.** *Biochem J* 2003, **374:**413-421.
2. Peters V, Rehm BH: **Protein engineering of streptavidin for in vivo assembly of streptavidin beads.** *Journal of biotechnology* 2008, **134:**266-274.
3. Jahns AC, Rehm BH: **Tolerance of the Ralstonia eutropha class I polyhydroxyalkanoate synthase for translational fusions to its C terminus reveals a new mode of functional display.** *Applied and environmental microbiology* 2009, **75:**5461-5466.
